# Impact of residential area on the management of rheumatoid arthritis patients initiating their first biologic DMARD: Results from the Ontario Best Practices Research Initiative (OBRI)

**DOI:** 10.1097/MD.0000000000015517

**Published:** 2019-05-17

**Authors:** Mohammad Movahedi, Raman Joshi, Emmanouil Rampakakis, Carter Thorne, Angela Cesta, John S. Sampalis, Claire Bombardier

**Affiliations:** a Ontario Best Practices Research Initiative, Toronto General Research Institute University Health Network, Toronto; b JSS Medical Research, St-Laurent, QC; c William Osler Health System, Brampton Civic Hospital, Brampton; d Southlake Regional Health Centre, Newmarket; e McGill University, Montreal, QC; f Department of Medicine (DOM) and Institute of Health Policy, Management, and Evaluation (IHPME), University of Toronto; g Division of Rheumatology, Mount Sinai Hospital, Toronto, Canada.

**Keywords:** biologic naïve, postal code, registry, residential area, rheumatoid arthritis, TNFi

## Abstract

Access to care and management of Rheumatoid Arthritis (RA) patients may differ based on residential area. We described differences in the profile of patients initiating their first biologic disease modifying antirheumatic drug (bDMARD) based on their residential area type.

Cross-sectional analysis of 793 adult RA patients in the longitudinal Ontario Best Practices Research Initiative (OBRI) registry initiating their first bDMARD <30 days prior to or anytime post-enrolment. Patient residential and clinic areas (rural vs. urban) were classified using 2 methods: postal codes and Statistics Canada population centres. Sociodemographics, disease characteristics, and RA medications (tumor necrosis factor inhibitor [TNFi] vs. non-TNFi, concurrent use of conventional synthetic DMARDs [csDMARDs], and intravenous [IV] vs. subcutaneous [SC] bDMARD) at initiation of first bDMARD were contrasted between residential area types.

Other than marital status, first language, and race (higher proportion of married, English speaking, Caucasian patients in rural areas), no significant differences were observed in the demographic and disease characteristics of patients living in rural and urban areas. In multivariate analysis, there was no association between residential area type and type of bDMARD use, concurrent csDMARD(s) use or route of bDMARD. However, patients living farther from their treating clinic were significantly less likely to initiate IV bDMARD. Female rheumatologist and rural clinic location were independently associated with lower odds of IV bDMARD use.

The use of SC vs. IV bDMARD was associated with being seen in a clinic located in a rural area, being treated by a female rheumatologist, and living farther from treating clinic. These results suggest possible prescription bias in bDMARD selection and/or patient preferences due to convenience.

## Abstract 2

Supplemental Digital Content is available in the text

## 1 Introduction

The introduction of biologic disease modifying antirheumatic drugs (bDMARDs) over the past 2 decades has improved RA outcomes. However, access to care and the management of rheumatoid arthritis (RA) patients may differ based on residential area which, in turn, can affect the evaluation of real-world effectiveness of antirheumatic medications.

Studies from different countries have shown that disease outcomes, burden of disease, and even disease prevalence may be associated with socioeconomic status, race/ethnicity, and geographic region.^[1–8]^ These differences may reflect limited access to care, services, and medications such as biologic treatments for populations within specific socioeconomic, regional, and ethnicity/race groups. There is a knowledge gap of understanding for this disparity.

In the present study, we aimed to describe differences in the profile of patients initiating their first bDMARD based on their residence in urban versus rural areas. We were also interested in investigating the association between residential area type and patient management in terms of type of first bDMARD selected, concurrent use of conventional synthetic disease modifying antirheumatic drugs csDMARD(s), and administration route of bDMARD.

## 2 Methods

### 2.1 Data source and patients

The Ontario Best Practices Research Initiative (OBRI) is a provincial registry that prospectively gathers long-term information on patients with RA followed in routine care. It incorporates rheumatologist assessments from approximately one-third of rheumatologists in the province of Ontario and a unique method of collecting data from the patients directly using telephone interviewers. Patients are eligible if they were ≥ 16 years of age at the time of diagnosis ≥ 18 years of age at enrolment, have a rheumatologist confirmed RA diagnosis, and have at least one swollen joint. Patients are recruited at any stage of disease and are managed as per the medical judgment of their rheumatologist.

Institutional research ethics approval was obtained prior to recruitment (REB#: 07–0729 AE).

### 2.2 Inclusion and exclusion criteria

**Figure 1:**
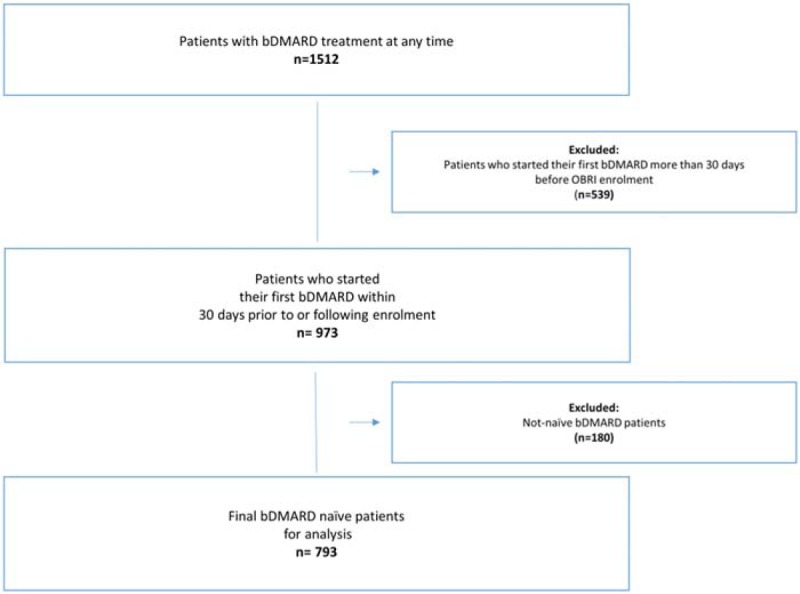
Cohort selection flow chart. bDMARD: biologic disease modifying antirheumatic drug.

Patients were included in the analysis if they had a clinical diagnosis of RA and had initiated treatment with a bDMARD within 30 days prior to enrolment in the OBRI registry or at any time following enrolment. Patients were excluded if they had biologics previously (Fig. 1).

### 2.3 Clinical and patient reported data

The clinical data collected during rheumatologist visits included: rheumatoid factor (RF) status, patient global assessment (PtGA), physician global assessment (PhGA), 28-tender joint count (TJC-28), 28-swollen joint count (SJC-28), the presence of erosion, and RA medication use including csDMARD(s), bDMARD(s), nonsteroidal anti-inflammatory drugs (NSAIDs), and oral steroids. Patient reported data collected by interviewers included: sociodemographic characteristics including residential address, health assessment questionnaire disability index (HAQ-DI), health assessment questionnaire pain index (HAQ-PI), fatigue score, and comorbidity profile.

### 2.4 Residential area definition

**Figure 2:**
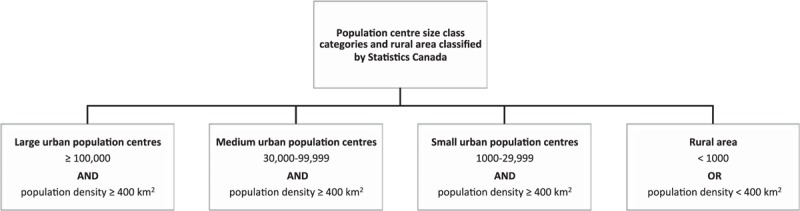
Population centre size categories.

Patient residential area type (rural vs. urban) was classified using 2 methods: Based on the forward sortation area (FSA) digit of the postal code of patients’ residence;^[9]^ Based on population centres as classified by Statistics Canada (2016) (Fig. 2).^[10]^

Additionally, we calculated the distance (in kilometres) between postal codes of patients’ residence and the treating clinic.

### 2.5 Outcome definition

Three different outcomes were assessed and their association with residential area type was examined: type of first bDMARD, according to the mechanism of action, defined as tumor necrosis factor inhibitors (TNFi) versus non-TNFi; concurrent use of csDMARD(s) at bDMARD initiation; type of administration route of first bDMARD, intravenous (IV) versus subcutaneous (SC).

#### 2.5.1 Statistical analysis

Descriptive statistics including the mean and standard deviation for continuous variables and counts and proportions for categorical variables were produced. The *t*-test or Wilcoxon rank test, as appropriate, were used for the comparison of residential area groups for continuous variables and the chi-square or the Fisher's exact test, as appropriate, for categorical variables.

To examine the independent association of patient residential area type and of the distance between patient residence and the treating clinic with the 3 different outcomes, a 2-step approach was followed where potential confounders were first identified based on whether they reached significance (*P* < .05) in the univariate logistic regression analysis. These potential confounders, along with age and gender, were then adjusted for in multivariate logistic regression analysis.

#### 2.5.2 Sensitivity analyses

To deal with missing data in the multivariate analyses, multiple imputations by chained equations was performed for missing variables and analyses were repeated for the full dataset as a sensitivity analysis. All variables in the final models were included in the imputation model. Twenty datasets were imputed and results were combined using Rubin's rules.^[11,12]^ In addition, the impact of clustering effect within clinical sites was examined by including site as a covariate in the regression models.

All statistical analyses were conducted using SAS 9.4 (SAS Institute, Cary, NC).

## 3 Results

A total of 793 biologic naïve RA patients were included in the analysis, of whom 128 (16.1%) resided in rural areas (based on the postal code method). The proportion of patients living in rural areas was higher when population centres were used (264 out of 761; 34.7%). Overall, the study cohort was comparable to the overall OBRI registry in terms of baseline characteristics (Table A1; Appendix).

Using both methods to classify rural/urban areas, there were no significant differences between residential area types in baseline sociodemographics except higher frequencies of married status (*P* < .001), Caucasian race (*P *< .001), and English spoken language (*P* < .001) in rural areas. Concurrent use of oral steroids was significantly lower in patients from urban areas based on postal code classification (20.0% vs. 27.3%, *P *= .04) (Table 1).

**Table 1:**
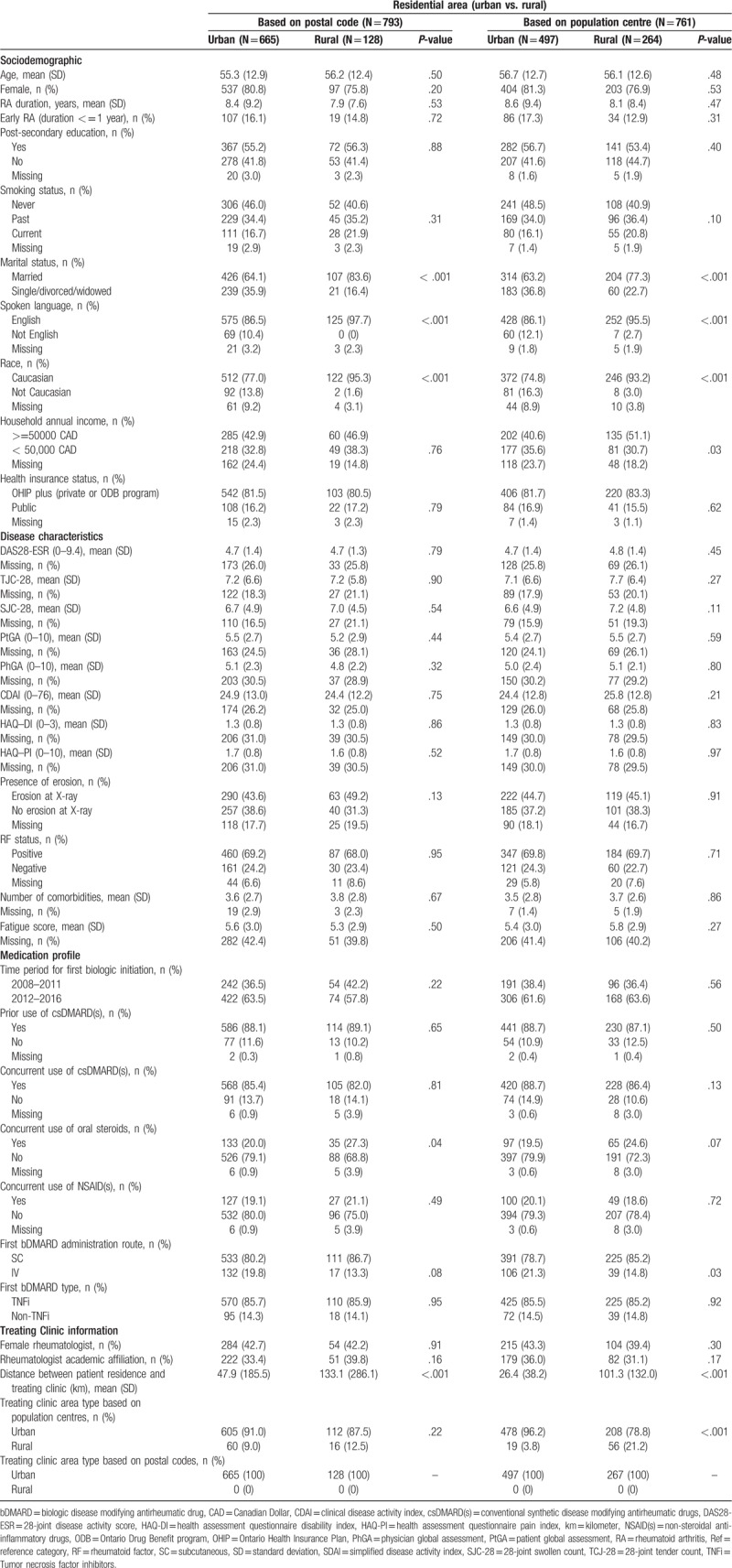
Baseline characteristics of biologic naïve patients according to residential area type as defined by postal code and population centre.

### 3.1 Impact of residential area on type of first biologic used by mechanism of action

Table 2 shows the results for the impact of residential area type on type of first bDMARD (TNFi vs. non-TNFi) using univariate and multivariate logistic regression models. Neither longer distance between patient residence and treating clinic address (model 1; _adj_OR [95%CI]: 1.01 [0.98–1.02], *P *= .80) nor living in a rural area (model 2; _adj_OR [95%CI]: 1.04 [0.50–2.16], *P *= .91 and model 3; _adj_OR [95%CI]: 0.68 [0.38–1.22], *P *= .20) had a significant impact on the type of bDMARD used. However, patients with a longer duration of RA disease, higher HAQ-DI and being initiated bDMARD in time period of 2012 to 2016 were significantly less likely to be initiated TNFi compared to non-TNFi. In contrast, patients on NSAID(s) and being treated by a clinical site located in rural area (based on population centres) were significantly more likely to start TNFi than non-TNFi (Table 2).

**Table 2:**
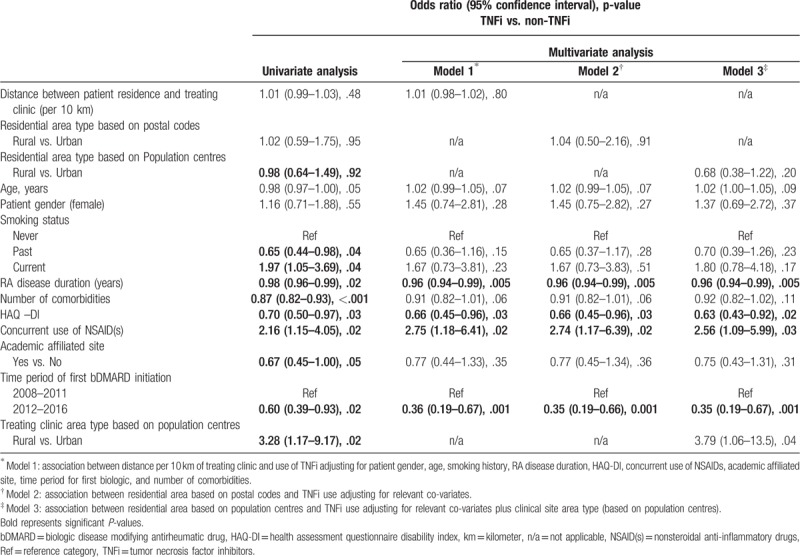
Impact of residential area type on type of first bDMARD; univariate and multivariate logistic regression analysis.

### 3.2 Impact of residential area type on concurrent use of csDMARD(s) with first bDMARD

Table 3 shows the results for the impact of residential area type on concurrent use of csDMARD(s) with the first bDMARD (combination therapy vs. bDMARD monotherapy). Neither longer distance between patient residence and treating clinic address nor living in a rural area had a significant impact on concurrent use of csDMARD(s). In all 3 models, higher number of comorbidities were significantly associated with a lower likelihood of concurrent csDMARD(s) use with the first bDMARD. In contrast, use of concurrent NSAID(s) was significantly associated with a higher likelihood of concurrent csDMARD(s) use.

**Table 3:**
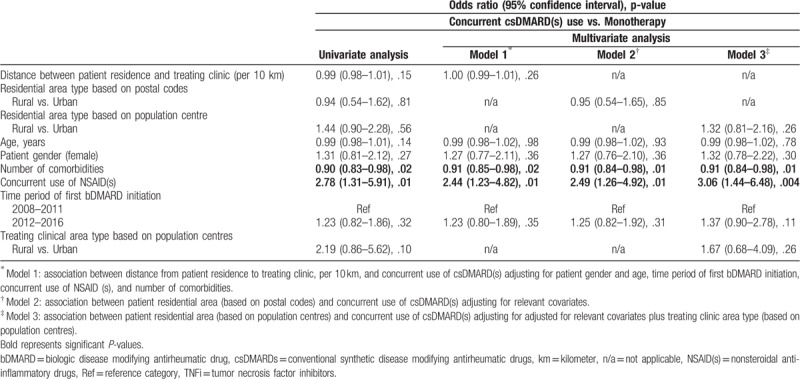
Impact of residential area type on concurrent use of csDMARD(s) with first bDMARD; univariate and multivariate logistic regression analysis.

### 3.3 Impact of residential area on administration route of first biologic

Table 4 summarizes the results of the univariate and multivariate logistic regression analyses assessing the impact of residential area type on administration route of first bDMARD. Patients living farther from their treating clinic (_adj_OR: 0.96; 0.93–0.99, *P *= .03) and in rural areas (model 2: _adj_OR: 0.64; 0.35–1.16, *P *= .14 and model 3: _adj_OR: 0.88; 0.56–1.39, *P *= .58) were less likely to be treated with IV bDMARD; however, for the residential area type the difference did not reach statistical significance. Treating clinics located in rural areas (based on population centres) (model 3: _adj_OR: 0.06; 0.01–0.41, *P *= .005), concurrent use of NSAID(s), female rheumatologists (all 3 models) and patient being a current smoker (model 2 and model 3) were associated with significantly lower odds of prescribing IV bDMARD compared to SC bDMARD. In contrast, patients with longer disease duration (all 3 models) and being initiated bDMARD in time period of 2012–2016 (model 2 and model 3) were significantly more likely to be prescribed a biologic agent that required infusion (Table 4).

**Table 4:**
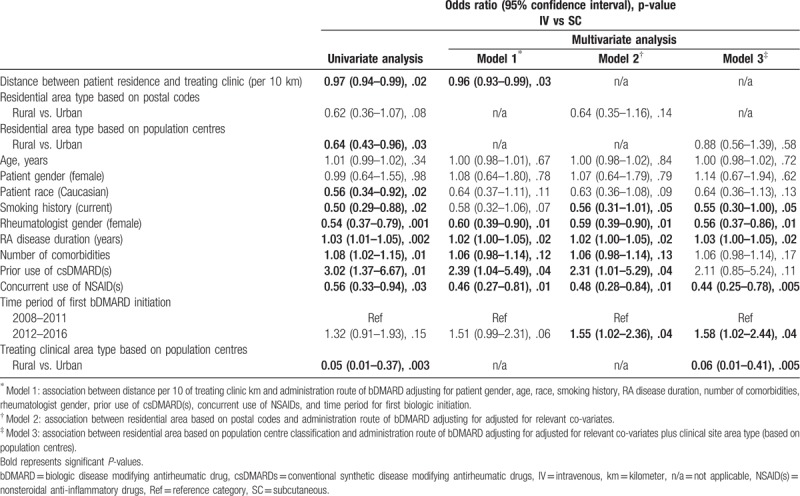
Impact of patient residential area type on administration route of first bDMARD; univariate and multivariate logistic regression analysis.

### 3.4 Sensitivity analyses

Repeating all analyses with a full dataset using multiple imputation, as sensitivity analysis, did not change the estimates for the impact of patient residential area or of the distance between the patient residence and the treating clinic on the type of bDMARD used, concurrent use of csDMARD(s), of the use of IV vs. SC bDMARD (Tables A2–A4; Appendix). Accounting for the cluster effect within each site also did not have an impact on the observed results.

## 4 Discussion

In the current study, we have explored whether patient residence in rural versus urban areas, as well as living distance from the treating clinic, had an impact on patient management among patients initiating their first bDMARD.

In addition to differences in sociodemographic characteristics, a higher RA disease activity as measured by swollen joint counts, although not statistically different, was observed in patients living in rural areas. Iltchev et al^[4]^ showed that RA morbidity in rural inhabitants of Poland was higher than in urban inhabitants. Bernatsky et al^[2]^ and Neovius et al^[5]^ compared the prevalence of RA between rural and urban areas. Hurd et al,^[8]^ in a systematic review, found most studies showed that indigenous patients with RA had higher disease activity and reported more significant impact on patient-reported outcomes and quality of life than non-Indigenous patients. Brekke et al^[3]^ found no significant differences in joint count scores and patient or rheumatologist evaluation of disease severity when comparing RA patients living in an affluent versus a less affluent area in the same city. However, to our knowledge, neither study directly compared sociodemographic and disease characteristics between patients residing in urban versus rural areas.

In terms of selection of bDMARD type or use of concurrent csDMARD(s), no differences were observed between patients living in rural and urban residential areas. Even though such decisions regarding patient management were not studied previously, Desai et al^[13]^ did show that initiation of TNFi (compared with not starting TNFi) differed significantly by geographic region in the United States. In contrast, Saag et al^[14]^ reported that there was no significant impact of urban/rural residence on physician visits for arthritis care.

We found that patients living farther from their treating rheumatologist's practice were more likely to be treated with SC as opposed to intravenous bDMARD which may be related to patient preferences and convenience. Furthermore, lower odds of treatment with intravenous bDMARD were observed in clinical sites located in rural areas; these results are expected as such sites may not have the required facilities and supporting healthcare staff for delivering infusions. Finally, female rheumatologists were less likely than males to prescribe intravenous bDMARDs over SC; although the exact reason behind this finding cannot be assessed one could speculate that it may relate to general factors influencing rheumatologists’ prescription such as subjective judgment and experience of the drugs, age and years in practice, or more consideration to patients’ preferences, among others.^[15]^

The fact that most patients in our study were assessed and treated at sites defined as urban and that, their care is provided mostly by trained/certified rheumatologists, may contribute to the relative homogeneity of results. Strengths of the current study include examining a large real-world RA patient population without strict inclusion criteria and no requirements for high disease activity which make it generalizable to routine clinical practice. In terms of limitations, there may be other unmeasured confounders which may have not been accounted for.

In summary, the use of SC versus IV medication was significantly associated with being seen in a clinic located in a rural area, being treated by a female rheumatologist, and living farther from treating clinic possibly reflecting prescription bias and patient preferences due to convenience, respectively. No other significant differences in the profile of RA patients or in patient management, in terms of bDMARD type and concurrent csDMARD use, were identified based on residential area type.

## Acknowledgments

The authors wish to knowledge OBRI-RA Investigators:

## Author contributions

Drs Ahluwalia, V., Ahmad, Z., Akhavan, P., Albert, L., Alderdice, C., Aubrey, M., Bajaj, S., Bensen, B., Bhavsar, S., Bobba, R., Bombardier, C., Bookman, A., Carette, S., Carmona, R., Chow, A., Ciaschini, P., Cividino, A., Cohen, D., Dixit, S., Haaland, D., Hanna, B., Haroon, N., Hochman, J., Jaroszynska, A., Johnson, S., Joshi, R., Kagal, A., Karasik, A., Karsh, J., Keystone, E., Khalidi, N., Kuriya, B., Larche, M., Lau, A., LeRiche, N., Leung, Fe., Leung, Fr., Mahendira, D., Matsos, M., McDonald-Blumer, H., Mittoo, S., Mody, A., Montgomery, A., Mulgund, M., Ng, E., Papneja, T., Pavlova, P., Perlin, L., Pope, J., Purvis, J., Rohekar, G., Rohekar, S., Ruban, T., Samadi, N., Shaikh, S., Shickh, A., Shupak, R., Smith, D., Soucy, E., Stein, J., Thompson, A., Thorne, C., Wilkinson, S.

All authors contributed in the conception or design of the work, revised the work critically and approved the final version of the manuscript.

**Conceptualization:** Mohammad Movahedi, Raman Joshi, Emmanouil Rampakakis, Carter Thorne, Angela Cesta, John S. Sampalis, Claire Bombardier.

**Data curation:** Raman Joshi, Carter Thorne, Claire Bombardier.

**Formal analysis:** Mohammad Movahedi.

**Methodology:** Mohammad Movahedi, Emmanouil Rampakakis, Angela Cesta, John S. Sampalis.

**Supervision:** Emmanouil Rampakakis, Claire Bombardier.

**Writing – original draft:** Mohammad Movahedi, Emmanouil Rampakakis, Angela Cesta.

**Writing – review & editing:** Mohammad Movahedi, Raman Joshi, Emmanouil Rampakakis, Carter Thorne, Angela Cesta, John S. Sampalis, Claire Bombardier.

## Supplementary Material

Supplemental Digital Content
