# Supplementary material for: Impact of residential area on the management of rheumatoid arthritis patients initiating their first biologic DMARD: Results from the Ontario Best Practices Research Initiative (OBRI)
Source: Medicine (Baltimore). 2019 May 17;98(20):e15517. doi: 10.1097/MD.0000000000015517 (PMC6531262; doi:10.1097/MD.0000000000015517)
Supplement: Supplemental Digital Content [file medi-98-e15517-s001.docx]

**Appendix**

**Table A1: Comparison of selected sociodemographic and disease profiles between study population and total OBRI registry**

|  | **Study cohort**  **based on postal code (n=793)** | **Study cohort**  **based on population centre (n=761)** | **Total OBRI**  **(n=3251)** |
| --- | --- | --- | --- |
| Age, mean (SD) | 56.3 (12.9) | 56.5 (12.7) | 57.6 (13.0) |
| RA duration, years, mean (SD) | 8.3 (9.0) | 8.4 (9.1) | 8.3 (9.7) |
| Female, n (%) | 634 (79.9%) | 607 (79.8%) | 2534 (78.0%) |
| Post-secondary education, n (%) | 439 (55.4%) | 423 (55.6%) | 1775 (54.5%) |
| Current or past smokers, n (%) | 413 (52.1%) | 400 (52.5%) | 1719 (52.8%) |
| Married, n (%) | 533 (67.2%) | 518 (68.1%) | 2158 (66.4%) |
| Caucasian race, n (%) | 634 (79.9%) | 618 (81.2%) | 2668 (82.1%) |
| Annual household income ≥ 50,000 CAD, n (%) | 345 (43.5%) | 337 (44.3%) | 1322 (41.0%) |
| DAS28 (0-9.4), mean (SD) | 4.7 (1.4) | 4.7 (1.4) | 4.3 (1.6) |
| CDAI (0-76), mean (SD) | 24.8 (12.8) | 24.9 (12.8) | 20.8 (13.7) |
| TJC-28, mean (SD) | 7.2 (6.5) | 7.3 (6.5) | 6.0 (6.3) |
| SJC-28, mean (SD) | 6.8 (4.9) | 6.8 (4.9) | 5.5 (5.0) |
| PtGA, mean (SD) | 5.4 (2.7) | 5.4 (2.7) | 4.8 (2.8) |
| PhGA (0-10), mean (SD) | 5.1 (2.3) | 5.1 (2.3) | 4.3 (2.5) |
| HAQ-DI (0-3), mean (SD) | 1.3 (0.8) | 1.3 (0.8) | 1.2 (0.8) |
| HAQ-PI (0-10), mean (SD) | 1.7 (0.8) | 1.7 (0.8) | 1.4 (0.9) |
| Presence of erosion, n (%) | 353 (44.5%) | 341 (44.8%) | 1272 (39.1%) |
| Number of comorbidities, mean (SD) | 3.7 (2.7) | 3.7 (2.7) | 2.3 (1.8) |
| Fatigue score, mean (SD) | 5.5 (3.0) | 5.5 (3.0) | 4.9 (3.1) |
| RF positive, n (%) | 547 (69.0%) | 531 (69.8%) | 2176 (67.0%) |
| Prior use of csDMARD(s), n (%) | 700 (88.3%) | 671 (88.2%) | 2818 (86.7%) |
| Concurrent use of steroid(s), n (%) | 168 (21.2%) | 162 (21.3%) | 823 (25.3%) |
| Concurrent use of NSAID(s), n (%) | 154 (19.4%) | 149 (19.6%) | 708 (21.8%) |

**Table A2: Impact of residential area type on type of first bDMARD; multivariate logistic regression analysis after multiple imputation**

|  | **Odds ratio (95% confidence interval), p-value**  **TNFi vs Non-TNFi** | | |
| --- | --- | --- | --- |
|  | Multivariate analysis | | |
|  | Model 1^©^  n=793 | Model 2 ^β^  n=793 | Model 3 ^€^  n=761 |
| **Distance between patient residence and treating clinic (per 10 km)** | 1.01 (0.99-1.03), .55 | n/a | n/a |
| **Patient residential area type based on postal codes** |  |  |  |
| - Rural vs. Urban | n/a | 1.01 (0.57-1.78), .97 | n/a |
| **Patient residential area type based on population centers** |  |  |  |
| - Rural vs. Urban | n/a | n/a | 0.78 (0.50-1.24), .30 |

©Model 1: association between distance per 10 km of treating clinic and use of TNFi adjusting for patient gender, age, smoking history, RA disease duration, HAQ-DI, concurrent use of NSAID(s), academic affiliated site, time period for first biologic, and number of comorbidities

**^β^**Model 2: association between residential area based on postal codes and TNFi use adjusting for relevant co-variates

^€^Model 3: association between residential area based on population centres and TNFi use adjusting for relevant co-variates plus clinical site area type (based on population centres)

Bold significant p-values.

TNFi: Tumour necrosis factor inhibitors; HAQ-DI: health assessment questionnaire disability index; NSAID(s): non-steroidal anti-inflammatory drugs; bDMARD: biologic disease modifying antirheumatic drug; n/a: not applicable

**Table A3: Impact of patient residential area on concurrent use of csDMARDs with first bDMARD; multivariate logistic regression analysis after multiple imputation**

|  | **Odds ratio (95% confidence interval), p-value**  **Concurrent csDMARD(s) use vs. Monotherapy** | | |
| --- | --- | --- | --- |
|  | Multivariate analysis | | |
|  | Model 1^©^  n=793 | Model 2 ^β^  n=793 | Model 3 ^€^  n=761 |
| **Distance between patient residence and treating clinic (per 10 km)** | 1.00 (0.98-1.01), .27 | n/a | n/a |
| **Patient residential area type based on postal codes** |  |  |  |
| - Rural vs. Urban | n/a | 0.95 (0.54-1.66), .85 | n/a |
| **Patient residential area type based on population centers** |  |  |  |
| - Rural vs. Urban | n/a | n/a | 1.33 (0.82-2.14), .25 |

©Model 1: association between distance from patient residence to treating clinic, per 10 km, and concurrent use of csDMARD(s) adjusting for patient gender and age, time period of first bDMARD initiation, concurrent use of NSAID (s), and number of comorbidities.

**^β^**Model 2: association between patient residential area (based on postal codes) and concurrent use of csDMARD(s) adjusting for relevant covariates

**^€^**Model 3: association between patient residential area (based on population centres) and concurrent use of csDMARD(s) adjusting for adjusted for relevant covariates plus treating clinic area type (based on population centres)

Bold significant p-values.

csDMARD(s): conventional synthetic disease modifying antirheumatic drugs; TNFi: Tumour necrosis factor inhibitors; NSAID(s): non-steroidal anti-inflammatory drugs; bDMARD: biologic disease modifying antirheumatic drug; n/a: not applicable

**Table A4: Impact of residential area type on administration route of first bDMARD; multivariate logistic regression analysis after multiple imputation**

|  | **Odds ratio (95% confidence interval), p-value**  **IV vs SC** | | |
| --- | --- | --- | --- |
|  | Multivariate analysis | | |
|  | Model 1^©^  ^n=793^ | Model 2 ^β^  ^n=761^ | Model 3 ^€^  ^n=761^ |
| **Distance between patient residence and treating clinic (per 10 km)** | **0.97 (0.94-0.99), .03** | n/a | n/a |
| **Patient residential area type based on postal codes** |  |  |  |
| - Rural vs. Urban | n/a | 0.67 (0.38-1.18), .17 | n/a |
| **Patient residential area type based on population centers** |  |  |  |
| - Rural vs. Urban | n/a | n/a | 0.85 (0.55-1.31), .46 |

©Model 1: association between distance per 10 of treating clinic km and administration route of bDMARD adjusting for patient gender, age, race, smoking history, RA disease duration, number of comorbidities, rheumatologist gender, prior use of csDMARD(s), concurrent use of NSAID(s), and time period for first biologic initiation.

**^β^**Model 2: association between residential area based on postal codes and administration route of bDMARD adjusting for adjusted for relevant co-variates

**^€^**Model 3: association between residential area based on population centre classification and administration route of bDMARD adjusting for adjusted for relevant co-variates plus clinical site area type (based on population centres)

Bold significant p-values.

csDMARD(s): conventional synthetic disease modifying antirheumatic drugs NSAID(s): non-steroidal anti-inflammatory drugs; bDMARD: biologic disease modifying antirheumatic drug; n/a: not applicable
